# Robotic versus laparoscopic pancreaticoduodenectomy for pancreatic and periampullary tumors: a meta-analysis

**DOI:** 10.3389/fonc.2024.1486504

**Published:** 2024-11-19

**Authors:** Gang Tang, Fang Chen, Rui Chen, Rongxing Zhou, Jingyi Zhang

**Affiliations:** ^1^ Division of Biliary Tract Surgery, Department of General Surgery, West China Hospital, Sichuan University, Chengdu, Sichuan, China; ^2^ Department of Medical Ultrasound, West China Hospital, Sichuan University, Chengdu, China

**Keywords:** robotic pancreaticoduodenectomy, laparoscopic pancreaticoduodenectomy, periampullary tumor, morbidity, meta-analysis

## Abstract

**Objective:**

The value of robotic pancreaticoduodenectomy (RPD) compared with laparoscopic pancreaticoduodenectomy (LPD) for pancreatic and periampullary tumors is controversial. This study aims to assess the available literature and compare the short outcomes of RPD and LPD.

**Methods:**

The PubMed, Cochrane Library, Embase, and Web of Science databases were searched to identify available research published up to 24 July, 2024. Risk ratios (RRs) and mean differences (MDs) with 95% confidence intervals (CIs) were calculated.

**Results:**

Seventeen studies with a total of 9417 patients (RPD group: 3334 patients; LPD group: 6083 patients) were included in this meta-analysis. The RPD group had lower overall morbidity (RR, 0.79), conversion (RR, 0.29) and blood transfusion rates (RR, 0.61), shorter length of stay (MD, -0.72 days), and higher number of harvested lymph nodes (MD, 0.62) than the LPD group. There were no significant differences in 90-day mortality (RR, 0.89), major complications (RR, 0.87), operative time (MD, -3.74 mins), blood loss (MD, -24.14 mL), reoperation (RR, 0.94), bile leak (RR, 0.62), postoperative pancreatic hemorrhage (RR, 0.96), postoperative pancreatic fistula (RR, 0.74), delayed gastric emptying (RR, 1.24), and R0 resection (RR, 1.00) between the groups.

**Conclusions:**

Compared with LPD, RPD for pancreatic and periampullary tumors could be safe and effective, and it has superior surgical outcomes. Further randomized controlled trials to verify the potential advantages of RPD over LPD are necessary.

**Systematic review registration:**

https://www.crd.york.ac.uk/PROSPERO/display_record.php?RecordID=581133, identifier CRD42024581133.

## 1 Introduction

Pancreaticoduodenectomy (PD) is a standard procedure for pancreatic and periampullary tumors (1, 2). PD is considered one of the most challenging procedures in hepatobiliary and pancreatic surgery due to the complex internal organ anatomy and digestive tract reconstruction required (1, 3). In recent years, despite improvements in surgical techniques and perioperative management, the postoperative complication rate of open PD remains as high as 46% in high-volume centers (4). Postoperative complications not only increase the economic burden of patients, but also damage the long-term survival of patients (5, 6).

Minimally invasive surgery (such as laparoscopic and robotic surgery) is a potential strategy to reduce perioperative morbidity due to less trauma, lower intraoperative blood loss and faster postoperative recovery (7). It has been widely used and offers proven advantages over open techniques in terms of short-term outcomes for various abdominal surgeries such as gastrectomy, colorectal surgery and prostate cancer surgery (8–11). Similarly, minimally invasive surgery has been increasingly used in pancreatic surgery in recent years. Compared to laparoscopic surgery, the robotic platform can provide more flexible operating instruments and a clearer and wider field of view (1). In theory, these advantages of robotic surgery could lead to better surgical outcomes. However, some recent clinical studies comparing robotic pancreaticoduodenectomy (RPD) and laparoscopic pancreaticoduodenectomy (LPD) have had conflicting results. The study by Farah et al. (12) showed that RPD has fewer complications and lower perioperative mortality compared to LPD. Zhang et al. ‘s study (13) included 2255 patients receiving PD, and the results showed no significant difference in postoperative morbidity and mortality between the RPD group and the LPD group. Unfortunately, systematic reviews and meta-analyses comparing the short-term outcomes of RPD and LPD in pancreatic and periampullary tumors are still lacking.

Therefore, we conducted a comprehensive collection of the currently published evidence and performed a meta-analysis to compare the efficacy and safety of RPD and LPD in the treatment of pancreatic and periampullary tumors. These results may help provide a valuable reference for surgeons in selecting surgical approaches.

## 2 Methods

### 2.1 Search strategy

This meta-analysis was follows the Preferred Reporting Items for Systematic Reviews and Meta-Analyses (PRISMA) (14). The study was registered in the PROSPERO database.

Two investigators independently conducted a comprehensive literature search using the Web of Science, PubMed, EMBASE, and Cochrane Library databases to identify studies published before 24 July, 2024. The details of the searching record were presented in **Table 1**. In addition, we checked the reference lists of the identified articles and related reviews to further screen for eligible studies. No language restrictions were applied during the search process.

**Table 1:** Electronic search strategy.

Database	Search term	Number
PubMed (Title/Abstract)	#1: da Vinci OR robot* OR robot-assisted OR robotic-assisted	#1: 82243
	#2: laparoscopy OR Laparoscop*	#2: 16122
	#3: pancreatoduodenectomy OR Pancreaticoduodenectom* OR Duodenopancreatectom* OR Whipple OR Whipple’s procedure OR Kausch-Whipple OR Kausch-Whipple procedure	#3: 18114
	#4: #1 AND #2 AND #3	#4: 314
Embase (Title Abstract Keyword)	#1: pancreatoduodenectomy OR Pancreaticoduodenectom* OR Duodenopancreatectom* OR Whipple’s procedure OR Kausch-Whipple OR Kausch-Whipple procedure	#1: 23167
	#2: Da Vinci OR Robot* OR Robot-assisted OR Robotic-assisted	#2: 118850
	#3: laparoscopy or Laparoscop*	#3: 264201
	#4: #1 AND #2 AND #3	#4: 561
Cochrane Library (Title Abstract Keyword)	#1: (Pancreatoduodenectomy) OR (Pancreaticoduodenectom*) OR (Duodenopancreatectom*) OR (Whipple’s procedure) OR (Kausch-Whipple) OR (Kausch-Whipple procedure)	#1: 1437
	#2: (Da Vinci) OR Robot* OR Robot-assisted OR Robotic-assisted	#2: 7976
	#3: laparoscopy OR Laparoscop*	#3: 28629
	#4: #1 AND #2 AND #3	#4: 29
Web of Science (Topic)	#1:(Da Vinci) OR (Robot*) OR (Robot-assisted) OR (Robotic-assisted)	#1: 558243
	#2: (laparoscopy) OR (Laparoscop*)	#2: 247721
	#3:(Pancreatoduodenectomy) OR (Pancreaticoduodenectom*) OR (Duodenopancreatectom*) OR (Whipple’s procedure) OR (Kausch-Whipple) OR (Kausch-Whipple procedure)	#3: 23551
	#4: #1 AND #2 AND #3	#4: 601

### 2.2 Study selection

Inclusion criteria were as follows (1): Patient: Patients diagnosed with pancreatic or periampullary (distal bile duct, ampulla, and duodenum) tumors (2); Intervention: RPD (3); Comparison: LPD (4); Outcomes: Primary outcomes encompassed 90-day mortality, overall morbidity, major complications, and length of stay. Secondary outcomes included blood loss, operative time, conversion, reoperation, bile leak, postoperative pancreatic fistula (POPF), postoperative pancreatic hemorrhage, delayed gastric emptying, blood transfusion, number of lymph nodes harvested, and R0 resection (5); Study type: randomized controlled trials (RCTs), cohort studies, and case-control studies.

The exclusion criteria were as follows: single-arm studies, animal studies, repeated publications, reviews, case reports, conference abstracts, and letters were excluded.

### 2.3 Data extraction

Data from all eligible studies were independently extracted by two investigators, and any disagreements were resolved by discussion with a third-party independent reviewer. The following data were extracted: author name, year of publication, country, study design, study population (sample size, age, and sex), and outcomes (90-day mortality, morbidity, length of stay, blood loss, operative time, conversion, reoperation, bile leak, POPF, postoperative pancreatic hemorrhage, delayed gastric emptying, blood transfusion, number of lymph nodes harvested, and R0 resection).

### 2.4 Quality assessment

The risk of bias in RCTs was assessed independently by two authors using the Cochrane risk-of-bias tool 2 (15) (1): randomization process (2), deviations from intended interventions (3), missing outcome data (4), measurement of the outcome (5), selection of reported results, and (6) overall risk of bias. For non-RCTs, the quality assessment was conducted independently by two authors using the Newcastle-Ottawa Scale (NOS), which assigns a score on a 9-point scale. A score of ≥7 indicates high quality, and scores of 5–6 indicate moderate quality. Any discrepancies were resolved through discussion, with intervention by a third author whenever necessary.

### 2.5 Statistical analysis

The meta-analysis was performed using the Review manager 5.3. Mean difference (MD) with corresponding 95% confidence intervals (CI) were calculated for quantitative data and risk ratios (RR) for qualitative variables. The I² statistic was used to assess the degree of statistical heterogeneity between included studies. A random-effects model was used if I² > 50%; otherwise, a fixed-effects model was employed (16). To explore the robustness of the results, we adopted the one-study exclusion method to evaluate the impact of each study on the total effect size. The potential publication bias was assessed using funnel plot and Egger’s tests if 10 or more studies were identified. Statistical significance was set at P value < 0.05.

## 3 Results

### 3.1 Literature retrieval

**Figure 1:**
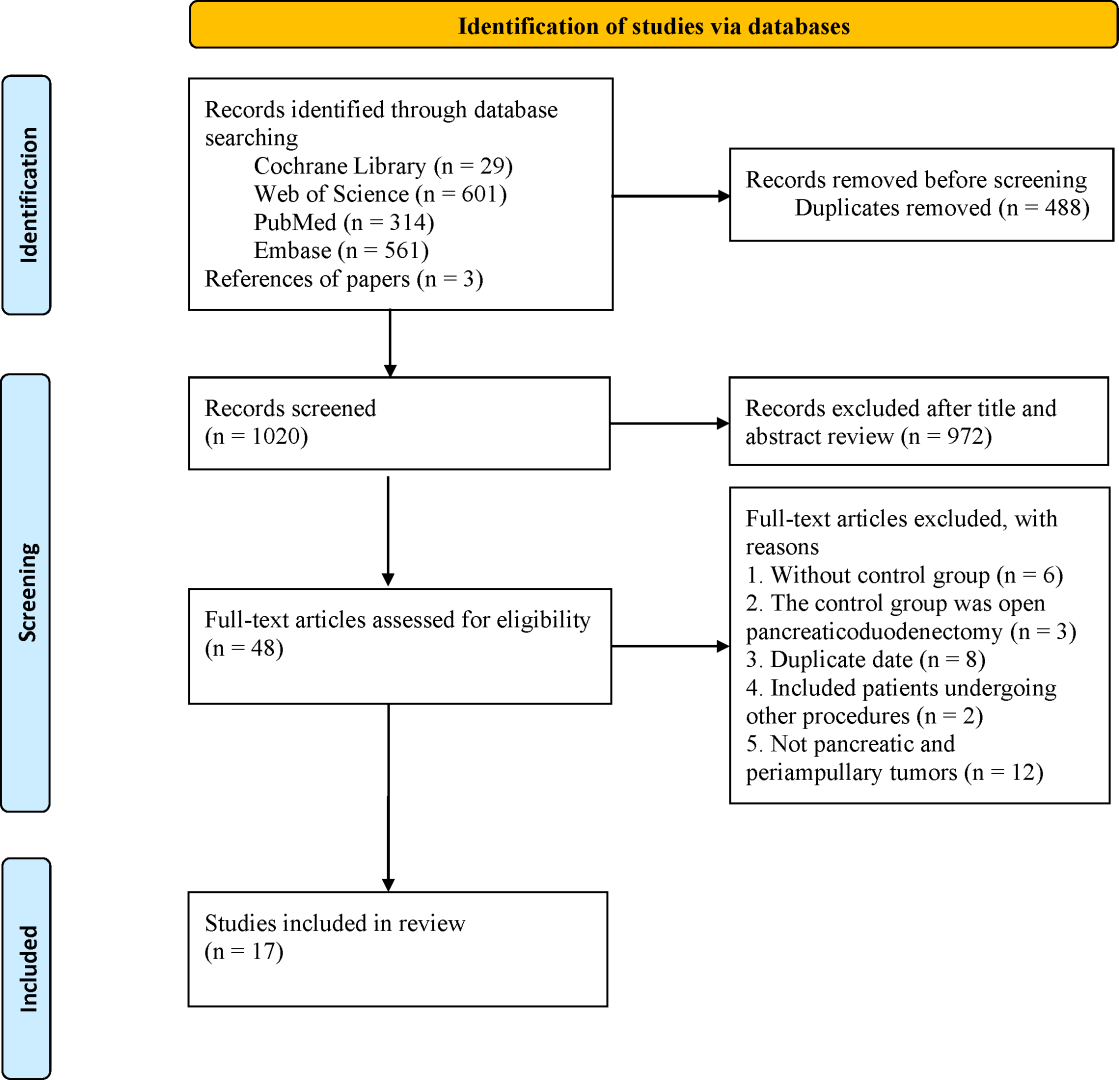
The PRISMA flowchart.

A total of 1508 articles were retrieved from four databases, and 488 duplicates were excluded. After reviewing titles and abstracts, 972 studies were excluded, and the full texts of the remaining 48 studies were evaluated. Finally, 17 studies (12, 13, 17–31) were included in the final analysis (**Figure 1**).

### 3.2 Study characteristics and quality assessment

The main characteristics of the included studies (12, 13, 17–30) are summarized in **Table 2**. The studies were published between 2016 and 2024 and included 15812 patients (RPD group: 3334 patients; LPD group: 6083 patients). Four of the studies adopted the PSM design (13, 23, 30, 31). The included patients were mainly from China (13, 17, 19, 22, 25, 29), the United States (23, 26, 30), Korea (21, 27, 31), UK (12, 20), Russia (24), and Singapore (18). All studies (12, 13, 17–31) were considered of moderate to high quality, achieving a score of ≥6 based on the NOS.

**Table 2:** Study Characteristics of the 17 included studies.

Author, year	Country	Period of study	Male	Study design	Age	Sample size	Robotic platforms	Included diseases	NOS
Liu 2017 (17)	China	2015-2016	RPD:14 LPD: 12	RCS	RPD: 57.16(68.56) LPD: 60.54(18.25)	RPD: 27 LPD: 25	The da Vinci^®^S Surgical System (Intuitive Surgical Inc., Sunnyvale, CA)	Periampullary neoplasms (9 benign lesions and 43 malignant lesions)	6/9
Goh 2019 (18)	Singapore	2014-2017	RPD:5 LPD: 16	RCS	RPD: 70(24-79) LPD: 62.5(24-79)	RPD: 10 LPD: 20	The da Vinci Si robotic platform	Periampullary tumours (7 benign lesions and 23 malignant lesions)	7/9
Zhang 2018 (19)	China	2013-2017	RPD:12 LPD: 11	RCS	RPD: 68(50-78) LPD: 64(42-76)	RPD: 20 LPD: 20	NA	Periampullary tumors (6 benign lesions and 34 malignant lesions)	7/9
Gall 2020 (20)	UK	2017-2019	RPD: 16 LPD: 19	RCS	RPD: 60.93(12.52) LPD: 65.18(11.36)	RPD: 25 LPD: 41	The Da Vinci Si and Xi models	Malignancies of the pancreatic head	7/9
Park 2021 (21)	Korea	2016-2020	RPD: 26 LPD: 30	RCS	RPD: 66.65(10.97) LPD: 65.70(12.97)	RPD: 49 LPD: 43	The da Vinci Xi Surgical System (Intuitive Surgical Inc., Sunnyvale, CA, USA)	Tumors confined to the pancreatic head or periampullary region (16 benign lesions and 76 malignant lesions)	8/9
Choi 2022 (31)	Korea	2012-2020	RPD: 26 LPD: 29	RCS, PSM	RPD: 60.02(11.97) LPD: 60.42(11.14)	RPD: 50 LPD: 50	NA	Periampullary tumors (44 benign lesions and 56 malignant lesions)	8/9
Guo 2022 (22)	China	2016-2020	RPD: 21 LPD: 12	RCS	RPD: 53.7(14.4) LPD: 52.1(13.5)	RPD: 32 LPD: 21	NA	Periampullary tumors (14 benign lesions and 39 malignant lesions)	6/9
Naffouje 2022 (23)	USA	2004-2017	RPD: 181 LPD: 553	RCS, PSM	RPD: 67.79(10.69) LPD: 67.86(10.31)	RPD: 358 LPD: 1074	NA	Stage I–III (T1–3 Nany M0) pancreatic adenocarcinoma	9/9
Tyutyunnik 2022 (24)	Russia	2007-2015	RPD: 43 LPD: 42	RCS	RPD: 62.5 (25–84) LPD: 62(34-82)	RPD: 100 LPD: 100	NA	Malignant and benign tumors of the head of the pancreas and periampullary area (57 benign lesions and 143 malignant lesions)	7/9
Zong 2022 (25)	China	2018-2022	RPD: 36 LPD: 77	RCS	RPD: 58.2(1.7) LPD: 58.1(1.4)	RPD: 76 LPD: 114	The Da Vinci Si Surgical System (Intuitive Surgical, Sunnyvale, CA, USA).	Periampullary benign or malignant tumors (42 benign lesions and 148 malignant lesions)	7/9
Kalabin 2023 (26)	USA	2010-2018	RPD: 347 LPD: 1390	RCS	RPD: 65.36(64.47-66.25) LPD: 64.97(64.55-65.39)	RPD: 676 LPD: 2677	NA	Pancreatic adenocarcinoma	7/9
Lee 2023 (27)	Korea	2015-2019	RPD: 10 LPD: 28	RCS	RPD: 57.7(11.6) LPD: 68.2(8.5)	RPD: 21 LPD: 60	NA	Distal bile duct cancer	7/9
Uijterwijk 2023 (28)	8 centers (6 in Europe, 1 in Australia, and 1 in Asia)	2010-2021	RPD: NA LPD: NA	RCS	RPD: NA LPD: NA	RPD: 37 LPD: 53	NA	Distal cholangiocarcinoma	6/9
Zhang 2023 (13)	China	2015-2022	RPD: 612 LPD: 622	RCS, PSM	RPD: 60.5(52.0-67.0) LPD: 61.0(52.0-67.0)	RPD: 1006 LPD: 1006	NA	Benign, premalignant, or resectable malignant or borderline resectable tumors of the pancreatic and periampullary region (383 benign lesions and 1629 malignant lesions)	9/9
Dai 2024 (29)	China	2016-2023	RPD: 27 LPD: 32	RCS	RPD: 59.8(10.6) LPD: 60.5(12.2)	RPD: 47 LPD: 54	The da Vinci Xi (or Si) Surgical System	Pancreatic cancer	8/9
Farah 2024 (12)	UK	2014-2021	RPD: NA LPD: NA	RCS	RPD: NA LPD: NA	RPD: 175 LPD: 100	NA	Pancreatic cancer	7/9
Wehrle 2024 (30)	USA	2010-2020	RPD: 323 LPD: 332	RCS, PSM	RPD: 66.5(10.4) LPD: 65.6(10.1)	RPD: 625 LPD: 625	NA	Pancreatic cancer	9/9

LPD, laparoscopic pancreaticoduodenectomy; NA, not available; PSM, propensity score matching; RCS, retrospective cohort study; RPD, robotic pancreaticoduodenectomy.

### 3.3 Meta-analysis

#### 3.3.1 90-day mortality

**Figure 2:**
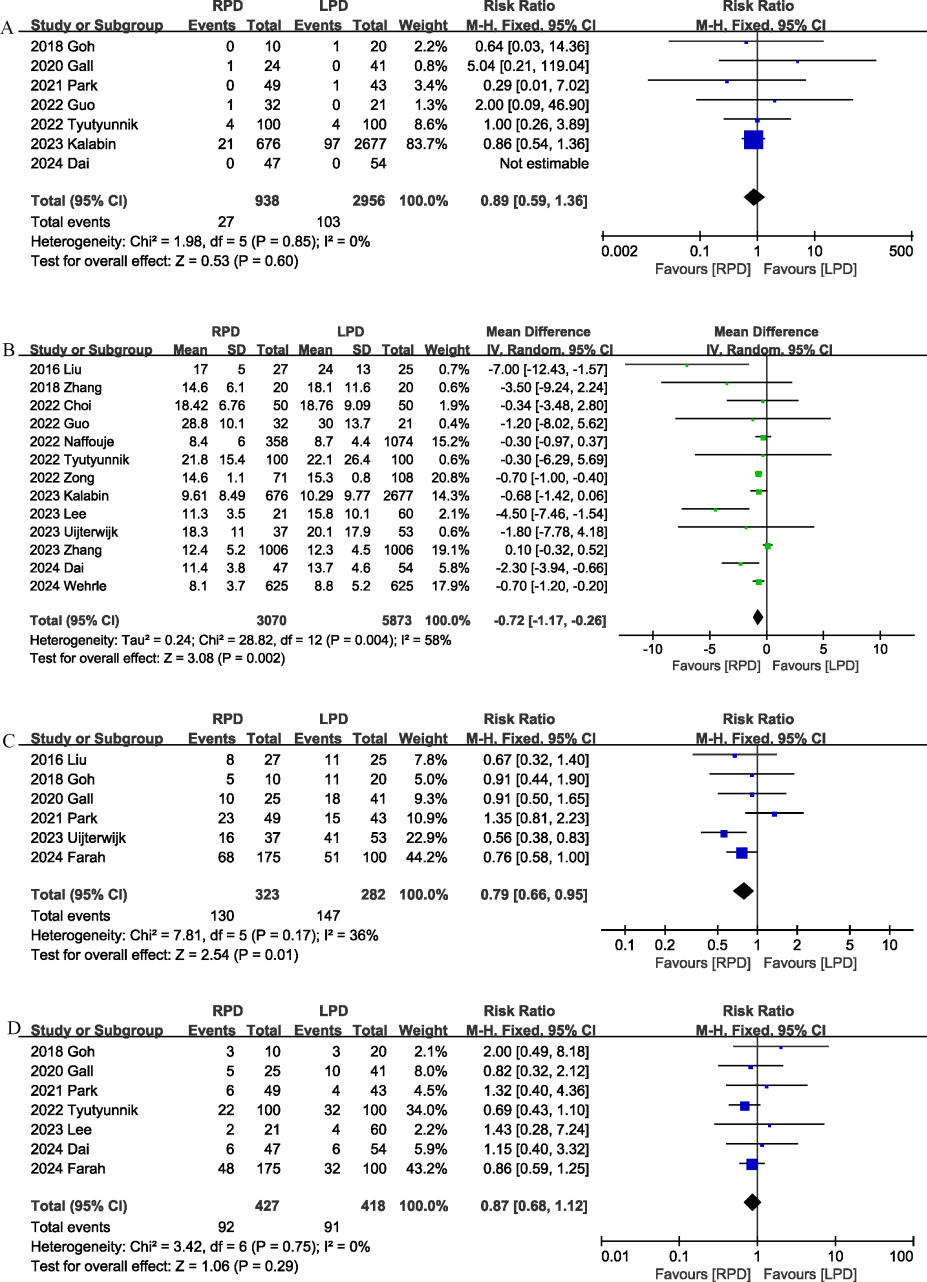
Comparison of primary outcomes between the two groups. **(A)** 90-day mortality, **(B)** length of stay, **(C)** overall morbidity, and **(D)** major complications.

Seven studies (18, 20–22, 24, 26, 29) reported data on 90-day mortality. The combined results of the 7 studies showed that there was no significant difference between the RPD group and the LPD group regarding this outcome with low heterogeneity (RR 0.89, 95% CI 0.59, 1.36; Heterogeneity: I^2^ = 0%, P = 0.85) (**Figure 2A**) (**Table 3**).

**Table 3:** Summary of results from all outcomes.

Outcomes	No. of studies	Events for RPD	Events for LPD	Effect size	95%CI	P	I^2^ (%)
Overall complications	6	130/323	147/282	0.79	0.66, 0.95	0.01	36
90-day Mortality	7	27/938	103/2956	0.89	0.59, 1.36	0.60	0
Major complications	7	92/427	91/418	0.87	0.68, 1.12	0.29	0
Postoperative pancreatic fistula	11	66/585	97/616	0.74	0.55, 1.00	0.05	0
Bile leak	8	22/383	39/424	0.62	0.37, 1.04	0.07	0
Delayed gastric emptying	8	95/526	63/503	1.24	0.67, 2.29	0.49	68
Postoperative pancreatic hemorrhage	5	25/216	28/219	0.96	0.57, 1.61	0.87	1
Blood transfusion	7	55/439	91/482	0.61	0.45, 0.83	0.002	27
R0 resection	6	717/896	2380/2957	1.00	0.93, 1.08	0.94	65
Reoperation	8	9/279	11/330	0.94	0.44, 2.02	0.87	0
Conversion	8	33/492	94/475	0.29	0.20, 0.42	<0.00001	31
Blood loss	13	–	–	-24.14	-55.98, 7.71	0.14	83
Operation time	11	–	–	-3.74	-22.74, 15.26	0.70	94
Number of lymph nodes harvested	8	–	–	0.62	0.28, 0.95	0.0003	36
Hospital stay	13	–	–	-0.72	-1.17, -0.26	0.002	58

#### 3.3.2 Length of stay

The length of the hospital stay was reported in 13 studies (13, 17, 19, 22–31). According to the results of this meta-analysis, RPD significantly reduced the length of hospital stay (MD, -0.72 days; 95% CI, -1.17, -0.26, P = 0.002) (**Figure 2B**).

#### 3.3.3 Morbidity

Six studies (12, 17, 18, 20, 21, 28) assessed overall complication. The pooled results suggested that RPD significantly reduced the overall complication rate (RR 0.79, 95% CI 0.66, 0.95, P = 0.01), with low heterogeneity (I^2^ = 36%, P = 0.17) (**Figure 2C**). Combined data from 7 studies (12, 18, 20, 21, 24, 27, 29) showed that the rates of major complications (Clavien–Dindo ≥ 3) were comparable between the RPD and LPD groups (RR 0.87, 95% CI 0.68, 1.12; Heterogeneity: I^2^ = 0%, P = 0.75) (**Figure 2D**).

#### 3.3.4 Blood loss

**Figure 3:**
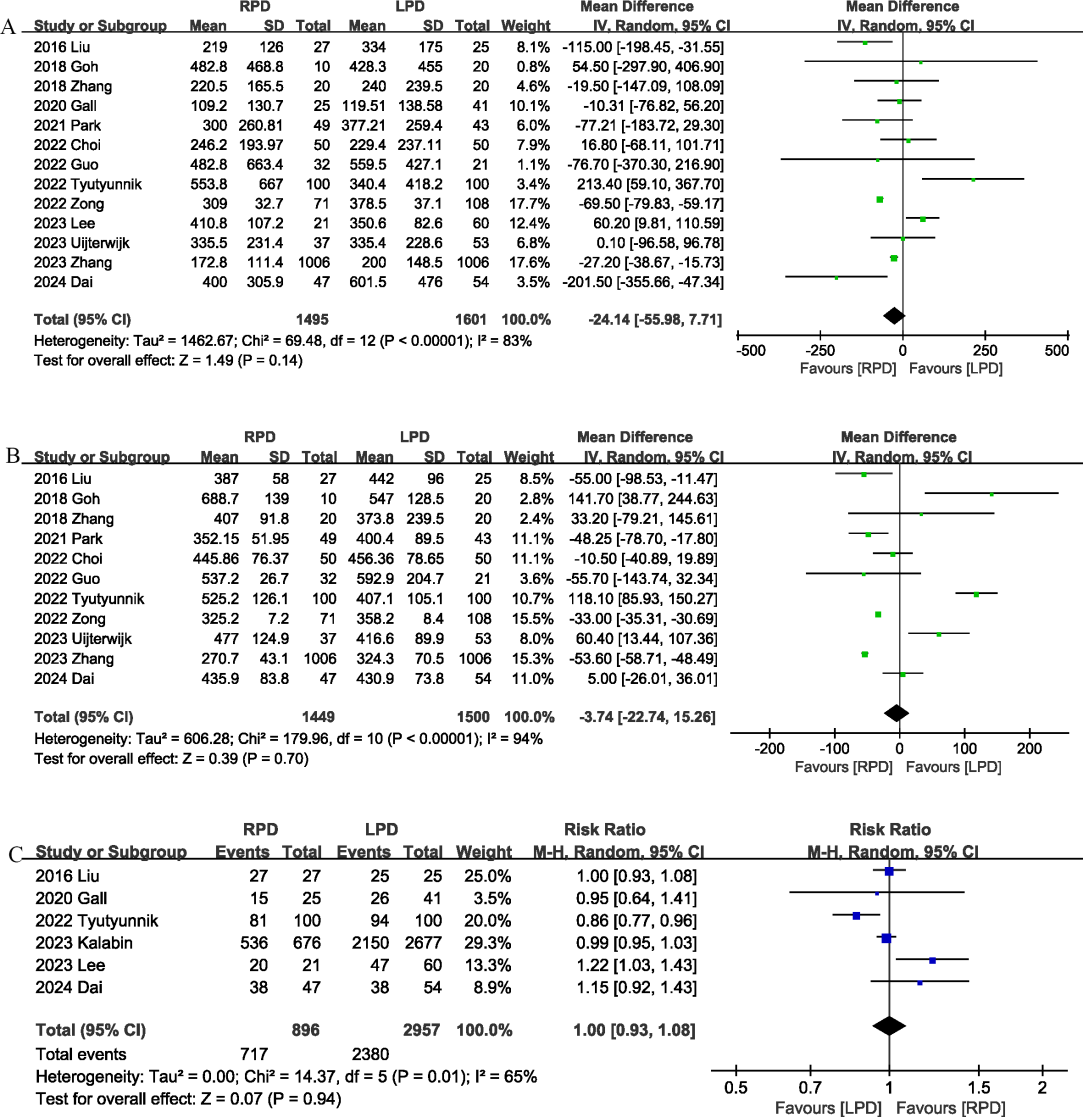
Comparison of secondary outcomes between the two groups. **(A)** intraoperative blood loss, **(B)** operative time, and **(C)** R0 resection.

Thirteen studies (13, 17–22, 24, 25, 27–29, 31) provided information on intraoperative blood loss. The combined results showed that the intraoperative blood loss was similar between the RPD group and the LPD group (MD, -24.14 mL; 95% CI, -55.98, 7.71, P = 0.14; I^2^ = 83%) (**Figure 3A**).

#### 3.3.5 Operation time

The operation time was reported in 11 studies (13, 17–19, 21, 22, 24, 25, 28, 29, 31). The combined results showed that the RPD group has similar operation time as compared with the LPD group (MD, -3.74 mins; 95% CI, -22.74, 15.26, P = 0.70) (**Figure 3B**).

#### 3.3.6 R0 resection

R0 resection was reported in 6 studies (17, 20, 24, 26, 27, 29), and the combined effect size suggested that the R0 resection rates were comparable between the two groups (RR 1.00, 95% CI 0.93, 1.08, P = 0.94; I^2^ = 65%) (**Figure 3C**).

#### 3.3.7 Number of lymph nodes harvested

**Figure 4:**
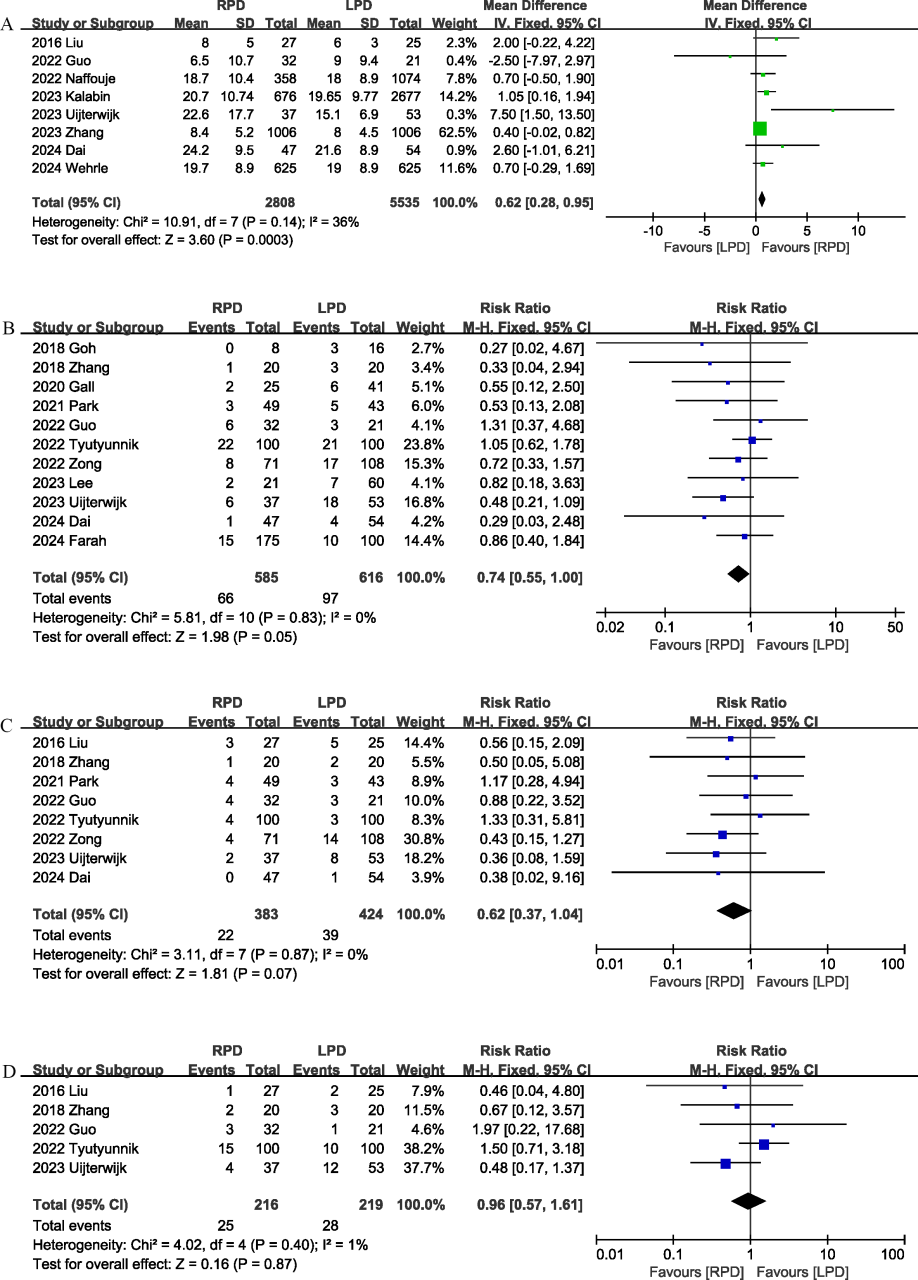
Comparison of secondary outcomes between the two groups. **(A)** number of lymph nodes harvested, **(B)** postoperative pancreatic fistula, **(C)** bile leak, and **(D)** postoperative pancreatic hemorrhage.

Eight trials (13, 17, 22, 23, 26, 28–30) reported the number of lymph nodes harvested. Compared with LPD, RPD significantly increased the number of lymph nodes harvested (MD, 0.62; 95% CI, 0.28, 0.95, P = 0.0003; I^2^ = 36%) (**Figure 4A**).

#### 3.3.8 Postoperative pancreatic fistula

Eleven studies (12, 18–22, 24, 25, 27–29) evaluated the POPF. There was no significant difference in the incidence of POPF (RR 0.74, 95% CI 0.55, 1.00, P = 0.05) (**Figure 4B**) between the RPD and LPD groups.

#### 3.3.9 Bile leak

Eight studies (17, 19, 21, 22, 24, 25, 28, 29) reported bile leaks. No significant differences were observed between the two groups (RR 0.62, 95% CI 0.37, 1.04, P = 0.07), and heterogeneity was low (I^2^ = 0%, P = 0.87) (**Figure 4C**).

#### 3.3.10 Postoperative pancreatic hemorrhage

Postoperative pancreatic hemorrhage was reported in 5 studies (17, 19, 22, 24, 28), and the combined effect size suggested that the postoperative pancreatic hemorrhage rates were comparable between the two groups (RR 0.96, 95% CI 0.57, 1.61, P = 0.87; I^2^ = 1%) (**Figure 4D**).

#### 3.3.11 Conversion rate

**Figure 5:**
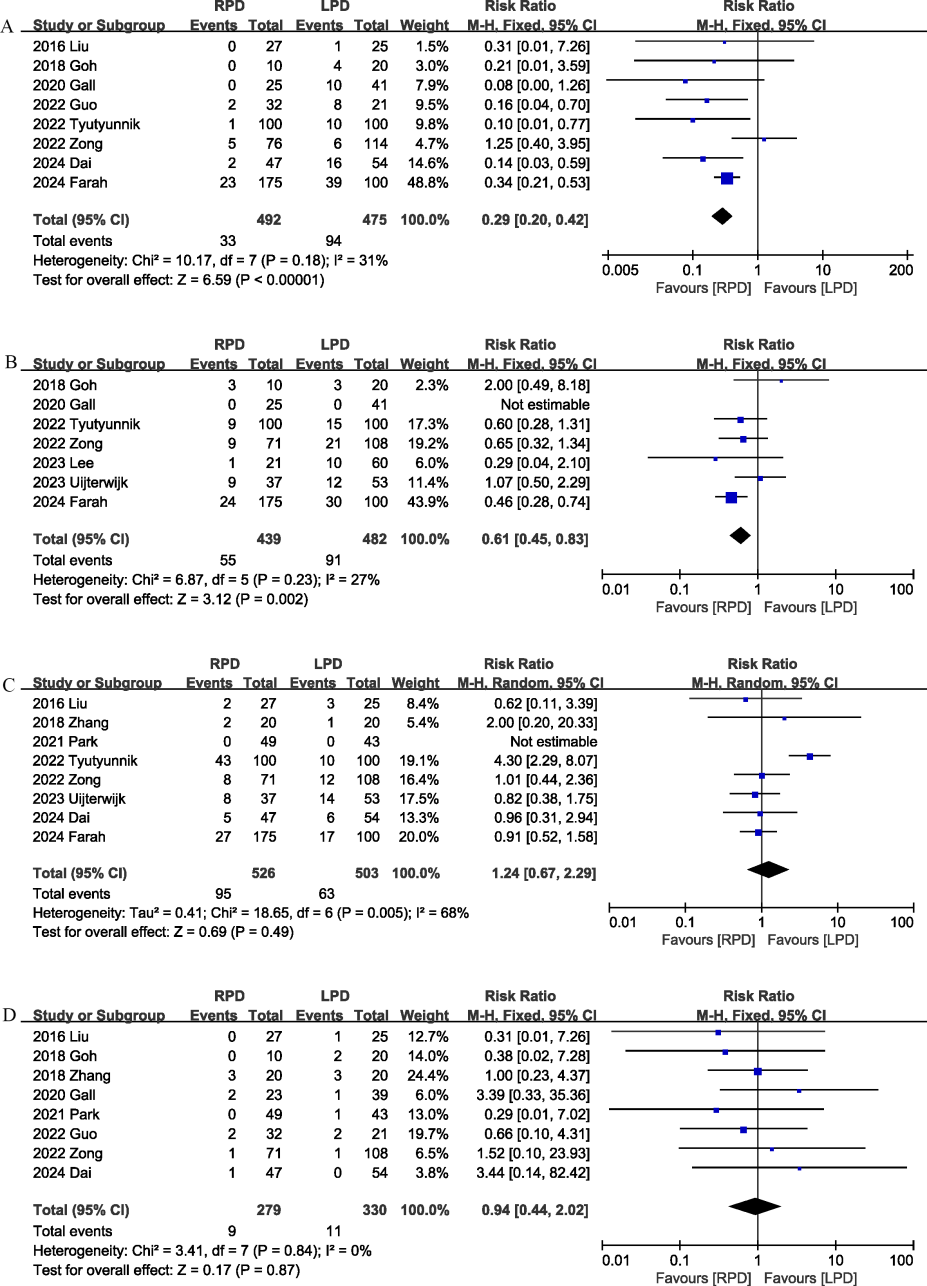
Comparison of secondary outcomes between the two groups. **(A)** Conversion rate, **(B)** blood transfusion, **(C)** delayed gastric emptying, and **(D)** reoperation.

Conversion rate was evaluated in 8 studies (12, 17, 18, 20, 22, 24, 25, 29), and the pooled results showed that RPD had lower conversion rate than LPD (RR 0.29, 95% CI 0.20, 0.42; heterogeneity: I^2^ = 31%, P = 0.18) (**Figure 5A**).

#### 3.3.12 Blood transfusion

Seven studies (12, 18, 20, 24, 25, 27, 28) compared blood transfusion rates between the RPD and LPD groups. The combined results showed that RPD was effective in reducing the blood transfusion rate (RR 0.61, 95% CI 0.45, 0.83, P = 0.002) (**Figure 5B**).

#### 3.3.13 Delayed gastric emptying

Delayed gastric emptying was reported in 8 studies (12, 17, 19, 21, 24, 25, 28, 29), and there was no significant difference in the incidence of delayed gastric emptying (RR 1.24, 95% CI 0.67, 2.29, P = 0.49) (**Figure 5C**) between the two groups.

#### 3.3.14 Reoperation

Eight trials (17–22, 25, 29) reported the reoperation rates. There were no significant differences between the two groups, and heterogeneity was low (RR 0.94, 95% CI 0.44, 2.02; Heterogeneity: I^2^ = 0%, P = 0.84; **Figure 5D**).

### 3.4 Publication bias and sensitivity analysis

**Figure 6:**
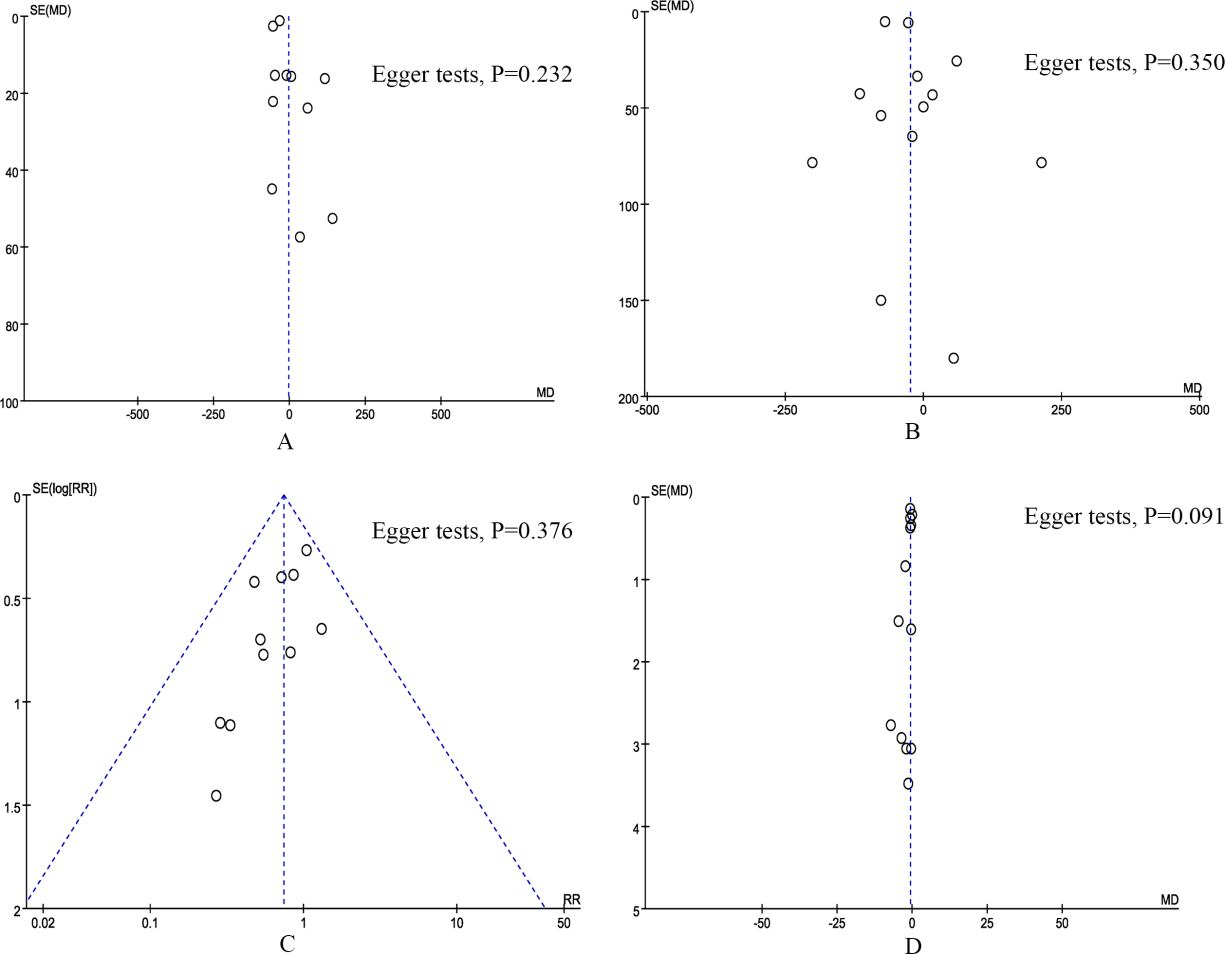
Funnel plot of primary outcomes. **(A)** operation time, **(B)** blood loss, **(C)** postoperative pancreatic fistula, and **(D)** length of stay.

According to the funnel plots and Egger tests (**Figure 6**), and no significant publication bias was observed for operation time, blood loss, POPF, and length of stay. Sensitivity analysis showed that no single study affected the overall effect size of the 90-day mortality, major complications, length of stay, conversion, reoperation, postoperative pancreatic hemorrhage, delayed gastric emptying, number of harvested lymph nodes, and R0 resection. The sensitivity analysis suggested that the total effect size of overall morbidity changed significantly when the study by Farah et al. (12) (RR 0.82, 95% CI 0.64, 1.04; I^2^ = 48%, P = 0.10) or the study by Uijterwijk et al. (28) (RR 0.86, 95% CI 0.71, 1.06; I^2^ = 7%, P = 0.37) was excluded. The total effect size of POPF changed significantly when the study by Guo et al. (22) (RR 0.72, 95% CI 0.53, 0.97; I^2^ = 0%, P = 0.83) or the study by Tyutyunnik et al. (24) (RR 0.64, 95% CI 0.45, 0.93; I^2^ = 0%, P = 0.92) was excluded. The total effect size of bile leak changed significantly when the study by Park et al. (21) (RR 0.57, 95% CI 0.33, 0.99; I^2^ = 0%, P = 0.89) or the study by Tyutyunnik et al. (24) (RR 0.56, 95% CI 0.32, 0.97; I^2^ = 0%, P = 0.92) was excluded. The total effect size of blood transfusion rates changed significantly when the study by Farah et al. (12) (RR 0.74, 95% CI 0.49, 1.10; I^2^ = 3%, P = 0.39) was excluded. The total effect size of operation time changed significantly when the study by Tyutyunnik et al. (24) (MD, -22.61 mins; 95% CI, -38.40, -6.82, P = 0.005) was excluded. The total effect size of blood loss changed significantly when the study by Lee et al. (27) (MD, -37.11 mL; 95% CI, -68.24, -5.99, P = 0.02; I^2^ = 79%) or the study by Tyutyunnik et al. (24) (MD, -32.39 mL; 95% CI, -62.94, -1.84, P = 0.04; I^2^ = 81%) was excluded.

## 4 Discussion

To our knowledge, this is the first meta-analysis to compare RPD and LPD for pancreatic and periampullary tumors. Based on evidence from 17 medium to high-quality studies, our meta-analysis showed that RPD significantly reduced overall postoperative complication rates, blood transfusion rates, and conversion rates, improved the number of lymph nodes harvested, and reduced length of hospital stay. In addition, there were no significant differences between RPD and LPD in postoperative mortality, major complication rate, reoperation rate, R0 resection rate, operative time, and blood loss. Our results have important clinical value because we provide evidence that short-term outcomes for RPD are not inferior to LPD, and this information may help surgeons select the appropriate surgical approach for patients with pancreatic and periampullary tumors.

The high morbidity and mortality after PD is an urgent problem for surgeons to solve. Minimally invasive surgical techniques offer a potential strategy for reducing postoperative morbidity in hepatobiliary and pancreatic surgery (32–34). LPD and RPD are two important minimally invasive procedures. Due to the shortcomings of laparoscopic surgery such as limited movement, unstable camera platform and two-dimensional imaging, LPD is more dependent on the surgical technique of the surgeon. Compared with laparoscopic surgery, robotic surgery retains the advantages of minimally invasive surgery, but also has a 3D visual surgical field of view and flexible operating instruments (6, 35). These advantages of robotic surgery may offer potential benefits in reducing complications after PD. This was also confirmed by our study, and our pooled results showed that RPD significantly reduced the total postoperative complication rate compared with LPD. In addition, in other surgical procedures, such as radical resection of rectal cancer and gastric cancer, similar results were seen in comparison of robotic surgery with laparoscopic surgery (36, 37). Pancreatic specific complications such as postoperative pancreatic hemorrhage, POPF and biliary fistula are common complications after PD, and are the main causes of perioperative death (2). Zhang et al.’s study (13) showed that POPF, PPH and reoperation were independent risk factors for postoperative mortality in PD. Consistent with the findings of several existing clinical studies (38–40), our meta-analysis suggests that the rates of these complications (postoperative pancreatic hemorrhage, POPF, and biliary fistula) for RPD and LPD are comparable. Similarly, several previous meta-analyses (41, 42) have shown no significant difference in pancreas-specific complications between the robotic approach and the laparoscopic approach in pancreatic surgery.

R0 resection and lymph node dissection are two important measures of oncology efficacy. R0 resection was associated with long-term survival (43). A meta-analysis of 61 studies including 62,529 patients by Kamarajah et al. (43) showed no significant difference in R0 resection rates between surgical approaches (RPD, LPD, and open PD). Similar results were observed in our study. Adequate lymph node dissection is essential for accurate staging (44). Malleo et al. (45) analyzed data from 1218 patients and showed that patients with pancreatic ductal adenocarcinoma need to obtain at least 28 lymph nodes. The RPD has an enlarged 3D field of view and a tremor filter that may aid in precise lymph node dissection (41). Our meta-analysis showed that RPD significantly increased the number of lymph nodes obtained in malignant. This is similar to a meta-analysis by Ouyang et al. (41), which included patients receiving PD for benign or malignant disease and showed that significantly more lymph nodes were obtained in the RPD group than in the LPD group.

A potential challenge to the popularity of RPD is the lengthening of surgical time. Some early studies (46–48) have shown that surgery time for RPD is significantly longer than for LPD. However, the results of this meta-analysis suggest that RPD does not extend the duration of surgery compared to LPD. This may be related to surgeons’ lack of experience with robotic surgery in earlier studies. Zhang et al. ‘s (13) study showed that when PD was performed by a surgeon who completed the RPD learning curve, the operation time in the RPD group was even shorter than that in the LPD group. The robotic platform provides enlarged images, reduces hand tremors, enables precise sutures, and may have potential advantages in reducing intraoperative blood loss. This study showed that although intraoperative blood loss was comparable between the RPD and LPD groups, the perioperative blood transfusion rate for RPD (12.5%) was significantly lower than for LPD (18.8%).

Conversion to open surgery is associated with poorer surgical outcomes. A recently published meta-analysis suggests that conversion to open in minimally invasive PD is associated with an increase in major postoperative complications and delayed postoperative recovery (49). Our meta-analysis showed that RPD had significantly lower transfer rates than LPD. Similarly, some previous meta-analyses (41, 50, 51) have shown that the conversion rate for hepatobiliary and pancreatic surgery is significantly lower in the robotic group than in the laparoscopic group. In addition, our study showed shorter hospital stays in the RPD group, which may be related to lower postoperative morbidity and conversion rates in the RPD group.

There are several limitations to our meta-analysis. First, the studies we included were all non-RCTs and were limited by the inherent limitations of retrospective studies. Second, there was a lack of evaluation of the long-term efficacy of RPD in the included studies. Considering the advantages of RPD in reducing postoperative morbidity and conversion rate and increasing the number of lymph nodes harvested, further evaluation of the difference in long-term prognosis between RPD and LPD is warranted. In addition, hospital volume may have an impact on surgical outcomes of RPD versus LPD. In our meta-analysis, some of the included studies included data from both high-volume and low-volume centers, so a subgroup analysis could not be performed to further evaluate the effect of hospital volume on the results. Future, high-quality studies with large samples to assess the effect of surgical center volume on the outcome of RPD versus LPD are warranted. Finally, there was high heterogeneity in some outcomes (operation time and blood loss), so these results should be treated with caution.

In conclusion, this meta-analysis showed that RPD can provide short-term perioperative outcomes that are not inferior to those of LPD in pancreatic and periampullary tumors. In addition, compared with LPD, RPD has potential advantages in reducing postoperative complications, blood transfusion and conversion to laparotomy, shortening hospital stay, and increasing the number of lymph nodes harvested. Further high-quality RCTs are necessary to demonstrate the benefits and clinical value of RPD.

## Data Availability

The original contributions presented in the study are included in the article/supplementary material. Further inquiries can be directed to the corresponding authors.
